# Poplar’s Waterlogging Resistance Modeling and Evaluating: Exploring and Perfecting the Feasibility of Machine Learning Methods in Plant Science

**DOI:** 10.3389/fpls.2022.821365

**Published:** 2022-02-11

**Authors:** Xuelin Xie, Xinye Zhang, Jingfang Shen, Kebing Du

**Affiliations:** ^1^College of Sciences, Huazhong Agricultural University, Wuhan, China; ^2^Hubei Academy of Forestry, Wuhan, China; ^3^College of Horticulture and Forestry Sciences, Hubei Engineering Technology Research Center for Forestry Information, Huazhong Agricultural University, Wuhan, China

**Keywords:** flood disaster, prediction of waterlogging tolerance, machine learning, feature selection, model establishment and evaluation

## Abstract

Floods, as one of the most common disasters in the natural environment, have caused huge losses to human life and property. Predicting the flood resistance of poplar can effectively help researchers select seedlings scientifically and resist floods precisely. Using machine learning algorithms, models of poplar’s waterlogging tolerance were established and evaluated. First of all, the evaluation indexes of poplar’s waterlogging tolerance were analyzed and determined. Then, significance testing, correlation analysis, and three feature selection algorithms (Hierarchical clustering, Lasso, and Stepwise regression) were used to screen photosynthesis, chlorophyll fluorescence, and environmental parameters. Based on this, four machine learning methods, BP neural network regression (BPR), extreme learning machine regression (ELMR), support vector regression (SVR), and random forest regression (RFR) were used to predict the flood resistance of poplar. The results show that random forest regression (RFR) and support vector regression (SVR) have high precision. On the test set, the coefficient of determination (R^2^) is 0.8351 and 0.6864, the root mean square error (RMSE) is 0.2016 and 0.2780, and the mean absolute error (MAE) is 0.1782 and 0.2031, respectively. Therefore, random forest regression (RFR) and support vector regression (SVR) can be given priority to predict poplar flood resistance.

## Introduction

Natural disasters are inherently a phenomenon that has adverse consequences for society (Paprotny et al., 2018). It damages the living environment and life of human beings. Flood disasters, as one of the most common and expensive natural disasters, have caused huge losses to human lives and property (Hu et al., 2018; Ao et al., 2020). With the development of social industry and economy, the warming of the atmosphere caused by greenhouse gas emissions may increase the risk of river flooding (Hallegatte et al., 2013; Hirabayashi et al., 2013; Willner et al., 2018; Bloeschl et al., 2019). Therefore, many studies want to build a system for predicting flood risk (Alfieri et al., 2017; Shafizadeh-Moghadam et al., 2018; Choubin et al., 2019; Khosravi et al., 2019), and a variety of machine learning methods are used in these studies. Choubin et al. (2019) used multivariate discriminant analysis (MDA), classification and regression trees (CART), and support vector machine (SVM) algorithms to predict flood risk in Iran’s Khiyav Chai drainage basin. The results show that the residential areas at the outlet of the drainage basin are very susceptible to floods. Khosravi et al. (2019) adopted three Multi-Criteria Decision-Making techniques (VIKOR, TOPSIS, and SAW) and two Machine Learning methods (NBT and NB) to test the flood sensitivity modeling of the Ningdu River Basin in China. Finally, their research shows that the NBT model is a powerful tool for evaluating flood-prone areas, and can properly plan and manage flood disasters. Nevertheless, predicting flood risk cannot substantially reduce the life and economic losses of human society. Afforestation can strengthen the stability of water, soil, and carbon sinks in the forest ecosystem, thereby effectively coordinating the relationship between humans and the natural environment. A considerable number of studies have shown that afforestation can weaken the impact of global warming and effectively reduce the risk of river flooding (Hong et al., 2018, 2020; Liu X. et al., 2018; Forster et al., 2021). Thus, afforestation is widely used to resist flood disasters.

Plants have evolved numerous resistance mechanisms to resist flood disasters, including plant morphological Screening of Candidate Genesristics, metabolic responses, and molecular transcriptional regulation (Loreti et al., 2016; Du et al., 2017; Yin et al., 2017; Zeng et al., 2019; Lukic et al., 2020; Lee et al., 2021). Among the diverse plant populations, poplar has become the main flood-resistant tree varieties in flood-prone areas due to its rapid growth and flood resistance features. Many studies have shown that the root system is the key organ of poplar responding to Flooding stress (Coleman et al., 2000; Major and Constabel, 2007; Berhongaray et al., 2013; Ye et al., 2018; Gerjets et al., 2021). Flooding stress affects the diffusion of oxygen in plant root tissues. At the same time, it limits the mitochondrial respiration of root cells and accumulates toxic substances, which seriously affects its normal physiological activities (Arbona et al., 2008; Voesenek and Bailey-Serres, 2013; Tian et al., 2019). In addition, flooding stress will destroy the photosynthesis performance of plants, which will inhibit plant growth and biomass accumulation (Du et al., 2012; Zhu et al., 2016; Zheng et al., 2017; Xiong et al., 2019; Zhou et al., 2020). Flooding stress not only reduces the chlorophyll content of plants, but also reduces the carotenoid content (Zhou et al., 2017). Kreuzwieser et al. (2009) found that the metabolite changes occurred in leaves and roots of submerged poplar. Du et al. (2012) compared the physiological and morphological adaptability of two poplar clones (hypoxia-resistant and hypoxia-sensitive) to flooding, and Peng et al. (2018) monitored the different response mechanisms of these two clones of poplar to flooding stress. These studies have greatly promoted people’s understanding of the waterlogging resistance mechanism, and to a considerable extent, strengthened people’s resistance to flood risks. Thus far, there are still few studies on the influence of poplar on the waterlogging resistance factors. These factors include the intrinsic features of poplar trees (photosynthesis and chlorophyll fluorescence, etc.) and external environmental features (ambient temperature, humidity, etc.). As a popular research direction, machine learning has recently been gradually introduced into the field of plant science. For the research on the resistance of poplar to waterlogging, Xie and Shen (2021) used poplar photosynthesis features and external environmental factors to predict the waterlogging tolerance of poplar. By using the SVR method in machine learning, they confirmed the feasibility of applying photosynthesis and other characteristic parameters to predict poplar flood resistance. However, previous prediction studies did not consider important parameters such as chlorophyll fluorescence. Additionally, the related forecasting research is not systematic enough, and the corresponding investigation and research are still lacking.

Based on the above considerations, the main purpose of this article is to consider more comprehensive feature parameters and use a variety of machine learning methods to predict the flood resistance of poplar. At the same time, it aims to supplement and improve the key content and procedures of poplar flood resistance prediction. First of all, the evaluation indicators of waterlogging tolerance were well defined and explained. Then, 26 internal characteristics and external environmental factors of poplars were screened by using feature selection algorithms such as significance test and stepwise regression. Finally, four machine learning methods were used to establish the flood resistance models of poplar, and the results were comprehensively evaluated in detail. Compared with previous studies, this study supplements the evaluation index and prediction system of poplar waterlogging tolerance. The main contribution is that the definition and analysis of evaluation indicators for waterlogging tolerance have been improved, and more comprehensive characteristic parameters have been considered. Moreover, the feature selection, prediction methods, and evaluation indicators were adjusted, and more machine learning methods and results have been considered and analyzed. This research has enriched the prediction of poplar’s flood resistance, which is of great significance to poplar’s accurate flood resistance, intelligent selection of seedlings, and cultivation of high-quality saplings. Furthermore, to a considerable extent, it promotes the research of flood resistance mechanisms, which have great theoretical and practical value.

## Materials and Methods

### Experimental Area and Materials

Research area: Huazhong Agricultural University, Wuhan, China (114°35′E, 30°49′E), subtropical humid monsoon climate. This area has four distinct seasons, with plenty of sunshine and plenty of rainfall. The annual average temperature is 15.8–17.5°C, rainfall is 1,269 mm, and total sunshine hours are between 1,810 h to 2,100 h.

Experimental objects: There were 20 poplar varieties in total. The scientific names corresponding to the 20 poplar varieties are shown in Table 1.

**TABLE 1:** Scientific names of 20 poplar varieties.

Varieties	Scientific names
LS68	*Populus deltoides “Lux” × P. simonii (LS68)*
LS81	*P. deltoides “Lux” × P. simonii (LS81)*
NL895	*P. × euramericana “Nanlin 895”*
I-63	*P. deltoides “Harvard”*
I-69	*P. deltoides “Lux”*
I-72	*P. euramaricana “an Martino”*
I-214	*P. × euramericana “I-214”*
I-45-51	*P. × euramaricana “I-45/51”*
Flevo	*P. euramericana “Flevo”*
Juba	*P. deltoides “55/56” × P. deltoides “2KEN8”*
LH04-13	*P. deltoides “Lux” × P. deltoides “Harvard” (LH04-13)*
LH04-17	*P. deltoides “Lux” × P. deltoides “Harvard” (LH04-17)*
Triplo	*P. euramericana “Triplo”*
DD102-4	*P. deltoides “DD102-4”*
Raspalje	*P. deltoides “Raspalje”*
Danhong	*P. deltoides “Danhong”*
Canadensis	*P. canadensis Moench.*
2L2025	*P. deltoides “Lux” × P. deltoides “Shanhaiguan”*
Ningshanica	*P. ningshanica*
Lushan	*P. × liaoningensis*

### Experimental Process and Parameter Measurement

The 1-year-old branches of 20 poplar clones were cut into about 15 cm cuttings with 3–4 buds. There were 4 experimental groups and 4 control groups for each variety, with a total of 160 experimental materials. After being soaked in water for 24 h, the cuttings were planted in mixed soil. The container was seedling pots (150 mm × 100 mm × 130 mm), and the soil was 1:1 substrate soil and peat soil (The soil consisted of 2–5% N, P_2_O_5_ and K_2_O, pH = 6.2, total organic matter of nutrient soil was ≥ 28%, and the total nutrient was ≥ 2%). The morphological changes of the plants were observed every day, including the chlorosis and shedding of leaves. We measured the height, biomass, photosynthesis, and chlorophyll fluorescence parameters of poplar seedlings on the 0th and 60th days. The characteristic parameters were measured by the LI-6400 photosynthesis analyzer (LI-COR, Lincoln, NE, United States), and the time was concentrated between 9:00 am and 11:30 am. In the experiment, a standard LI-COR leaf chamber and red and blue light sources (6400-02 LED light sources) were used. The light intensity was 1,000 μmol⋅m^–2^⋅s^–1^, and the air velocity was 500 μmol⋅s^–1^. 26 characteristic parameters of poplar samples were measured, including photosynthesis, chlorophyll fluorescence features, and environmental variables. The specific information of these features is shown in Appendix Table A1, and the treatment process of the experimental group and the control group is as follows.

• Control group: Watered normally (CK). There were drainage holes at the bottom of the flower pots in the Control group. Watered the plants according to the needs of normal plant growth, and the soil moisture was maintained at about 75% of the maximum water holding capacity in the field.
• Experimental group: Shallow flooded (FL). The waterlogging test was started 5–6 weeks after cuttings, and the water surface was 10 cm higher than the soil surface. The experiment lasted for 60 days, of which, the flooding time was 45 days, and the drainage recovery time was 15 days.

### Programming Environment

In this article, R 4.0.5 was used to perform data Processing and Feature selection process, and MATLAB R2018a was used to implement the Model building and evaluation.

## Methodology

**FIGURE 1:**
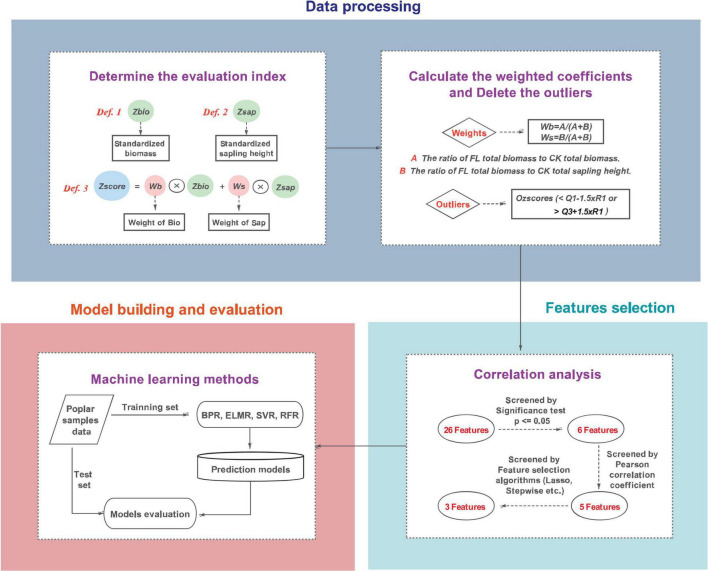
Flow chart of Methodology.

The methodology is divided into data processing, feature selection, model establishment and evaluation. The main procedures are shown in Figure 1, and the specific implementation steps will be introduced one by one below.

### Data Processing

#### Evaluation Index of Waterlogging Tolerance

The changes in biomass and seedling height can reflect the waterlogging tolerance of plants. In previous studies, Xie and Shen (2021) proposed the waterlogging tolerance evaluation index Zscore. This article supplemented the definition of the other two waterlogging tolerance evaluation indicators, and used the three waterlogging tolerance evaluation indicators for outlier analysis. Finally, the most suitable evaluation index for waterlogging tolerance was selected. The definitions of the three evaluation indicators are given below.

The first evaluation index for waterlogging tolerance is Zbio, which is obtained based on changes in biomass. This indicator is based on the change in biomass of the test group within 60 days to judge the flood resistance of poplar, and it is dimensionless. The stronger the waterlogging resistance performance, the larger the corresponding Zbio. The calculation method is shown in Formula (1):


(1) $$Zbio=\frac{bio\left(x_{i}\right)-E\left(bio\right)}{Std\left(bio\right)}$$

The second waterlogging tolerance evaluation index is Zsap, which is similar to the definition of Zbio. This index only considers the change of poplar seedling height, and its calculation method is shown in Formula (2):


(2) $$Zsap=\frac{sap\left(x_{i}\right)-E\left(sap\right)}{Std\left(sap\right)}$$

The third evaluation index of waterlogging tolerance is Zscore. This indicator takes into account the changes in biomass, as well as changes in seedling height. Compared with Zbio and Zsap, this index can more comprehensively reflect the flood resistance of poplar, and its calculation formula is shown in Formula (3):


(3) $$Zscore\left(x_{i}\right)=\omega_{bio}\times Zbio+\omega_{sap}\times Zsap$$

where ω_*bio*_ and ω_*sap*_ are the weight coefficients, which satisfy the condition ω_*bio*_ω_*sap*_ = 1. The calculation method of the weight is shown in equation (4). Zbio and Zsap are the two evaluation indicators mentioned in above.


(4) $$\omega_{bio}=\frac{A}{A+B},\omega_{sap}=\frac{B}{A+B}$$

where $A=\frac{FL(Sum\left(bio\left(x_{i}\right)\right)}{CK(Sum\left(bio\left(x_{i}\right)\right)},B=\frac{FL(Sum\left(sap\left(x_{i}\right)\right)}{CK(Sum\left(sap\left(x_{i}\right)\right)}$.

#### Treatment of Outliers

Extremely different from other observations, the outliers often cause anomalies (Aggarwal and Yu, 2005). Outliers may affect the accuracy of the final model (Domingues et al., 2018; Zhao et al., 2020). Consequently, before feature selection and models establishment, outliers in the data should be eliminated. The outlier Ozscore is defined in formula (5):


(5) $$\text{Ozscore}>Q_{3}+1.5\times R_{1}or\text{Ozscore}<Q_{1}-1.5\times R_{1}$$

where *Q*_3_ and *Q*_1_ are the upper and lower quartiles, and the quartile range *R*_1_ = *Q*_3_−*Q*_1_.

### Feature Selection

Feature selection is to effectively remove irrelevant and redundant features (Arora and Anand, 2019; Sayed et al., 2019). It can improve the performance of the model and reduce the cost of calculation (Li et al., 2018; Angulo and Shin, 2019). The 26 characteristic parameters considered in this study meet the conditions of multi-dimensional data. Therefore, these features need to be selected.

#### Hierarchical Clustering

Hierarchical clustering is a clustering method used to describe the hierarchical structure of samples in a group (Wu et al., 2009). The result of hierarchical clustering is usually represented by a dendrogram. The tree diagram shows the organization and relationship of the sample in the form of a tree, which is convenient for people to divide intuitively (Granato et al., 2018). For related clustering research work, refer to Xu and Wunsch (2005) and Murtagh and Contreras (2012). A hierarchical clustering method was adopted to cluster the poplar varieties and the five features selected by correlation analysis, and the measurement method was Euclidean distance.

#### Lasso and Stepwise Regression

The Lasso method is proposed by Tibshirani (1996) by combining the advantages of both ridge regression and subset selection meth. It not only has the interpretability of subset selection, but also has the stability of ridge regression. To achieve the purpose of feature selection, this method compresses the coefficients of insignificant variables to 0 (Zou and Hastie, 2005; Cui and Gong, 2018). Stepwise regression uses collinearity and variance contribution tests to gradually find all the significant features, thereby obtaining the optimal model. The basic idea of stepwise regression is to add new variables one by one, each time a new variable is added, consider whether to eliminate the selected variable until no more variables are introduced. Stepwise regression is mainly used to solve the problem of multicollinearity. For related research, refer to Guidolin and Pedio (2021), Ou et al. (2016), and Yang et al. (2019).

### Establishment and Evaluation of Regression Model

#### Machine Learning Methods

##### BP Neural Network

BP neural network is a multi-layer network structure composed of an input layer, an output layer, and one or more hidden layers (Yang et al., 2018), which can effectively deal with linear and non-linear relationships between data (Moghadassi et al., 2010). BP is called the error back propagation algorithm. In essence, the BP algorithm takes the error square as the objective function, and uses the gradient descent method to calculate the minimum value of the objective function. BP neural network can systematically solve the hidden layer connection weight learning problem of multilayer neural network, and it is one of the most widely used neural networks at present.

##### Extreme Learning Machine

The extreme learning machine is a new single hidden layer feedforward neural network (Ding et al., 2015). This algorithm can produce good generalization performance in most cases, and its learning speed is thousands of times faster than the traditional feedforward neural network algorithm. Therefore, many studies apply extreme learning machines for regression and prediction (Miche et al., 2010; Huang et al., 2011; Yao and Ge, 2018; Yaseen et al., 2018).

##### Support Vector Regression

Support vector machine (SVM) is a supervised machine learning method proposed by Cortes and Vapnik (1995) in the mid-1990s, which is used to deal with binary classification problems. The core idea of SVM is to find a hyperplane or hypersurface to segment the sample points to maximize the interval between the segmentation points. Support vector regression (SVR) is an application model of support vector machine (SVM) on regression problems (Demir and Bruzzone, 2014). As a classic regression algorithm in machine learning, support vector regression has been widely used in many fields, such as plant science, data mining, and biomedicine (Khosravi et al., 2018; Moazenzadeh et al., 2018; Zhuo et al., 2018; Han et al., 2019; Mishra and Padhy, 2019).

##### Random Forest Regression

Random forests produce reliable classifications by using predictions from a set of decision trees (Breiman, 2001). It is composed of multiple decision trees, and there is no correlation between each decision tree. The final output of the model is jointly determined by each decision tree in the forest. When dealing with regression problems, random forest uses the mean value of each decision tree as the final result. Due to the excellent regression results and the relatively fast processing speed, the use of random forest regression has also received extensive attention (Du et al., 2015; Chen et al., 2018; Dou et al., 2019).

The relationship between variables is often non-linear. Thus, compared with traditional linear regression, machine learning algorithms may have higher accuracy. There may be a non-linear relationship between poplar resistance to flooding and features. Consequently, the four machine learning methods mentioned above will be used to predict the waterlogging resistance of poplar.

#### Model Parameters

Manual tuning is the traditional method of adjusting the hyperparameters of machine learning models (Yang and Shami, 2020). With the improvement of automatic optimization methods, grid search (GS), particle swarm optimization (PSO), genetic algorithm (GA), and other optimization methods were proposed to find the optimal hyperparameters. to find the optimal hyperparameters. However, there are still problems such as complex optimization processes and slow convergence speed. Based on experience, we have selected the parameters of the four machine learning methods. The parameter sensitivity and parameter selection of each method will be analyzed below.

There are many kinds of training functions for the BPR algorithm, and most of the data sets are very sensitive to the training function. In the experiment, a variety of training functions were selected. Compared with other training functions such as trainlm function (based on Levenberg-Marquardt algorithm), the trainbr function based on Bayes rule has better network generalization ability and higher accuracy. Hence, the trainbr function was finally used in the BPR method. In addition, previous studies have shown that the number of hidden layer nodes is a key factor affecting the accuracy of BPR and ELMR models (Liu Z. T. et al., 2018; Zhang et al., 2018). For the number of hidden layer nodes, 3, 5, 7, 9, and 11 hidden layer nodes were used to train the BPR model, 2, 3, 4, 5, and 6 hidden layer nodes were used to train the ELMR model. The root mean square error (RMSE) of the training is shown in Table 2. When the hidden layer nodes of the BPR and ELMR methods were 9 and 5, respectively, the RMSE was considered to be the smallest. Therefore, the number of hidden layer nodes of BPR was set to 9, and the number of hidden layer nodes of ELMR was set to 5.

**TABLE 2:** RMSE of models with different Nodes or *Mtry*.

BPR		ELMR		RFR	
Nodes	RMSE	Nodes	RMSE	Mtry	RMSE
3	0.5654	2	0.3836	1	0.1940
5	0.4221	3	0.3785	2	0.2654
7	0.3703	4	0.3591	3	0.3210
9	0.3680	5	0.3426	4	0.3558
11	0.3939	6	0.3438	5	0.3982

For the SVR method, two SVR models (nu-SVR and epsilon-SVR) and four kernel functions (linear, polynomial, sigmoid, and radial basis functions) of the LibSVM toolbox were selected. Due to the higher precision of the model on the training set, the epsilon-SVR model (ε-SVR, a model that minimizes the RMSE) based on the RBF kernel function was finally selected. The regularization parameter C and the penalty coefficient gamma were determined by fivefold cross validation. The minimum number of leaves (*Mtry*) is the sensitive parameter of the RFR model, and the value of *Mtry* is generally set to 2 (Probst et al., 2018). In the experiment, we set the value of *Mtry* to 1, 2, 3, 4, and 5. The RMSE of the training function is shown in Table 2. When the value of *Mtry* was 1, RMSE was considered to be the smallest. Therefore, the value of *Mtry* was set to 1. Other parameters of the machine learning method were set as common parameters. The specific values of the parameters are shown in Table 3.

**TABLE 3:** Machine learning model parameters.

Methods	Model parameter
BPR	Training function: trainbr
	Number of input layers: 3
	Number of hidden layers: 9
	Number of output layers: 1
	Transfer function: logsig, purelin (Input-Hidden, Hidden-Output)
	net.trainparam.goal: 0.0001
	net.trainparam.lr: 0.01
	net.trainparam.epochs: 1000
ELMR	Training function: elmtrain
	Number of input layers: 3
	Number of hidden layers: 5
	Number of output layers: 1
	Activation function: sigmoid
SVR	Training function: svmtrain
	Model: ε-SVR
	Kernel function: RBF
	Regularization parameter C: 65
	Gamma: 0.001
	p: 0.01
RFR	Training function: TreeBagger
	Number of decision trees: 200
	Minimum number of leaves: 1
	Fraction of in-bag observations (FBoot): 1

#### Evaluation of Model Performance

The three evaluation indexes of coefficient of determination (R^2^), root mean square error (RMSE) and mean absolute error (MAE) were used to evaluate the performance of the model. The corresponding calculation formulas are shown in (6)-(8):


(6) $$R^{2}=1-\frac{\sum_{i=1}^{n}{\left(y_{i}-\hat{y_{\text{i}}}\right)}^{2}}{\sum_{i=1}^{n}{\left(y_{i}-\bar{y_{\text{i}}}\right)}^{2}}$$

(7) $$RMSE=\sqrt{\frac{\sum_{i=1}^{n}{\left(y_{i}-\hat{y_{\text{i}}}\right)}^{2}}{n}}$$

(8) $$MAE=\frac{1}{n}\sum_{i=1}^{n}\left|y_{i}-\hat{y_{\text{i}}}\right|$$

where *n* is the number of varieties, *y_i_* is the actual value, $\hat{y_{\text{i}}}$ is the predicted value, and $\bar{y_{\text{i}}}$ is the mean of the true *y_i_*.

## Results

### Treatment of Outliers and Selection of Evaluation Index

**FIGURE 2:**
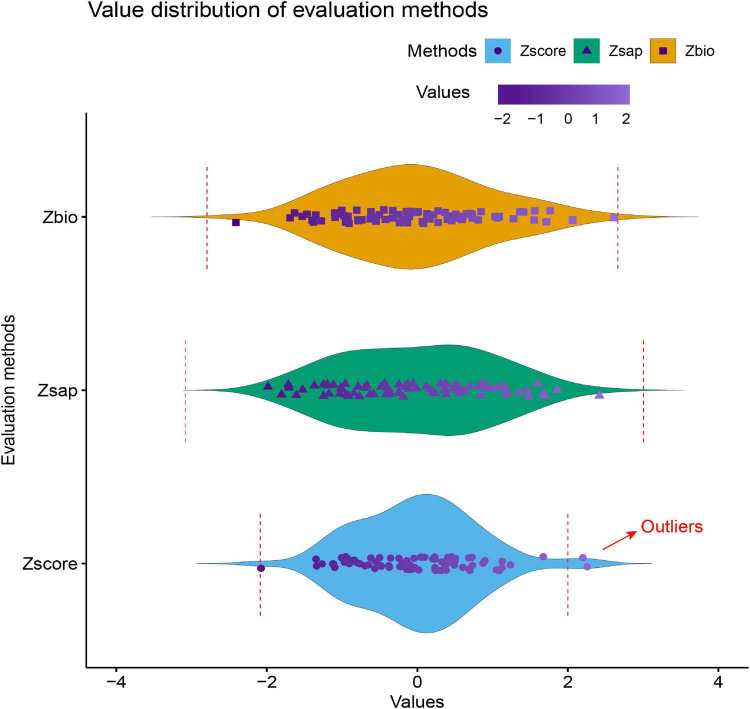
Distribution of three evaluation indexes.

Three evaluation indicators are used to deal with outliers in the data. The calculated descriptive statistics are shown in Table 4, where Max and Min are the maximum and minimum values, and Med is the median. The results of deleting outliers are shown in Figure 2.

**TABLE 4:** Descriptive statistics of the three evaluation indicators.

Methods	Min	Q_1_	Med	Q_3_	Max
Zbio	−2.409917	−0.748933	−0.095463	0.615642	2.614667
Zsap	−1.984857	−0.799594	−0.033385	0.722554	2.419308
Zscore	−2.076712	−0.554362	−0.039611	0.466923	2.257776

As shown in Figure 2, the points outside the red dotted line in the figure are outliers. It can be observed that for all samples, the defined Zscore roughly ranges from [−2, 2], while the ranges of Zbio and Zsap are larger than Zscore, and only Zscore has outliers. In addition, from the definition of the waterlogging tolerance evaluation index, we know that Zscore not only considers the biomass but also the change of seedling height, which can more comprehensively reflect the flood resistance of poplar. Thus, based on the above viewpoints, Zscore was finally selected as the waterlogging tolerance evaluation index in this article.

### Screening of Features

#### Significance Test and Correlation Analysis

**FIGURE 3:**
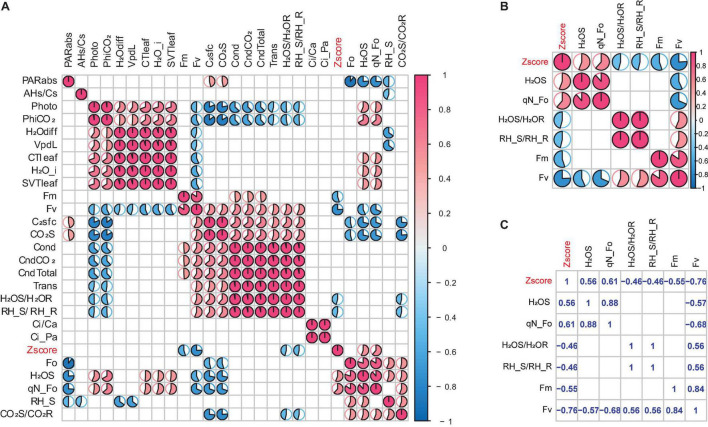
Heat map of Pearson’s correlation coefficient. **(A)** Heat map of 26 features. **(B)** Heat map of 6 features. **(C)** Specific values of 6 features.

According to the significance level of the correlation between the features and the poplar waterlogging tolerance score Zscores, 6 features were selected from 26 features, and these 6 features were all established under the condition that the significance level *p* = 0.05. We calculated the Pearson correlation coefficient, and the results are shown in Figure 3. Figure 3A is the heat map of 26 features, the blank part is the case of *p* = 0.05, that is, it is not significant. Figure 3B is a heat map of the correlation coefficient that satisfies the condition of *p* = 0.05, and Figure 3C is the exact value of Pearson’s correlation coefficient between 6 significant features. The specific meanings corresponding to the 6 significant features are shown in Appendix Table A1.

From Figure 3C, it can be found that the correlation between qN_Fo and H_2_OS is particularly strong. The correlation coefficient between them exceeds 0.8. Thus, the feature with the largest coefficient is selected from these related features, and the highly related features are excluded. After this operation, the retained features are Fv, qN_Fo, Fm, H_2_OS/H_2_OR, and RH_S/RH_R.

**FIGURE 4:**
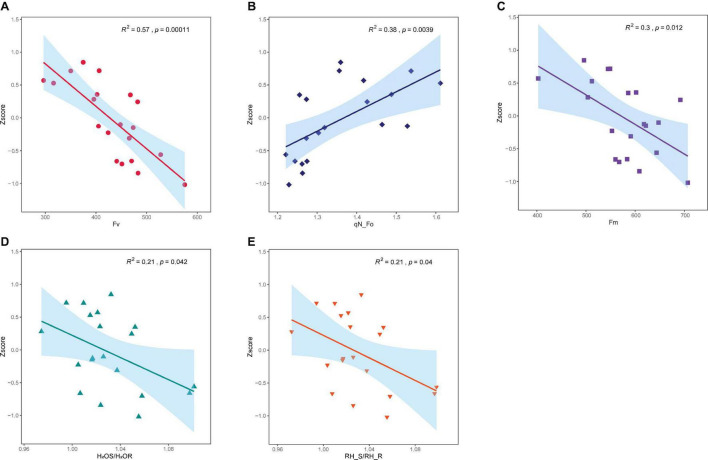
Regression prediction of single feature. **(A)** Fv. **(B)** qN_Fo. **(C)** Fm. **(D)** H_2_OS/H_2_OR. **(E)** RH_S/RH_R.

Before establishing the regression model, univariate regression prediction was carried out on the features of significance test and correlation screening, and the result is shown in Figure 4. It can be observed that the five variables all meet the significance level of *p* = 0.05, and there is a considerable proportional relationship between them. Nevertheless, the results of univariate regression were general, and the highest coefficient of determination (R^2^) is 0.57. For this reason, other methods should be chosen for regression analysis, such as multiple linear regression and machine learning regression methods.

It is undeniable that the 5 features of significance testing and correlation screening may still have multicollinearity. To implement machine learning modeling more reasonably and accurately, three methods of hierarchical clustering, Lasso, and Stepwise regression were adopted for further feature selection. Before predicting the waterlogging tolerance of different poplar varieties and further feature screening, the characteristic parameters and Zscore of each sample were averaged according to the variety.

#### Clustering Results

**FIGURE 5:**
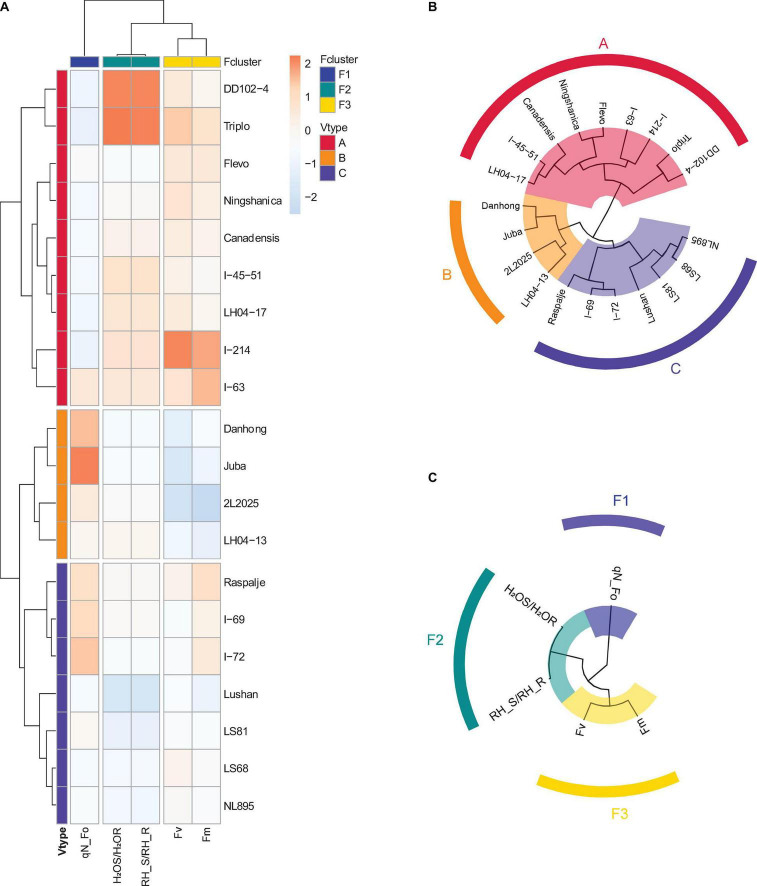
Results of poplar features and varieties clustering. **(A)** Total. **(B)** Varieties. **(C)** Features.

The results of hierarchical clustering are shown in Figure 5. Figure 5A is the total clustering heat map, Figure 5B is the poplar varieties clustering, and Figure 5C is the poplar characteristic clustering. According to the clustering results in Figure 5, we can divide poplar varieties and features into 3 groups. The classification of poplar varieties is marked as A, B, and C, and the classification of characteristic parameters is marked as F1, F2, and F3.

#### Results of Lasso and Stepwise Regression

Lasso regression and backward stepwise regression are used to screen the 5 features (Fv, qN_Fo, Fm, H_2_OS/H_2_OR, and RH_S/RH_R) obtained by significance and correlation. The results of the Lasso method are Fv, qN_Fo, and RH_S/RH_R. However, the screening result of stepwise regression only has the variable Fv. From the univariate regression analysis results in Figure 4, we know that the coefficient of determination (R^2^) of Fv is 0.57. A single feature used for regression may lack interpretability and may affect the accuracy of the final model. In addition, according to the results of hierarchical clustering in Figure 5, a feature with the largest correlation coefficient was selected from each of the three groups (F1, F2, and F3), and the results obtained are consistent with the Lasso method. Therefore, combining the results of hierarchical clustering and univariate analysis, in the final machine learning modeling, we used the three characteristic parameters of Fv, qN_Fo and RH_S/RH_R.

### Regression Results of Machine Learning Models

#### The Division of Test Set and Training Set

Before establishing the machine learning regression model, the poplar varieties were divided into training set and test set according to the ratio of 4:1 (the training set had 16 varieties, and the test set was 4 varieties). The four poplar varieties in the test set were selected from the three groups of A, B, C by stratified sampling based on the poplar hierarchical clustering results. The poplar varieties used and their corresponding Zscore and Vtype are shown in Table 5.

**TABLE 5:** Main information of poplar varieties.

Samples	Z score	V type
Canadensis	−0.31136376	A
DD102-4	−0.659417544	A
Flevo	−0.149084082	A
I-214	−1.018704692	A
I-63	0.244020869	A
LH04-17	0.348729466	A
Ningshanica	−0.843303666	A
Danhong	0.714574083	B
Juba	0.528127585	B
LH04-13	0.845992953	B
I-69	0.356889463	C
I-72	−0.12622992	C
LS68	−0.662543236	C
LS81	0.717527146	C
Lushan	0.282405203	C
NL895	−0.227444975	C
I-45-51	−0.702652584	A
Triplo	−0.561219766	A
2L2025	0.570264018	B
Raspalje	−0.103562886	C

#### Training Set

**FIGURE 6:**
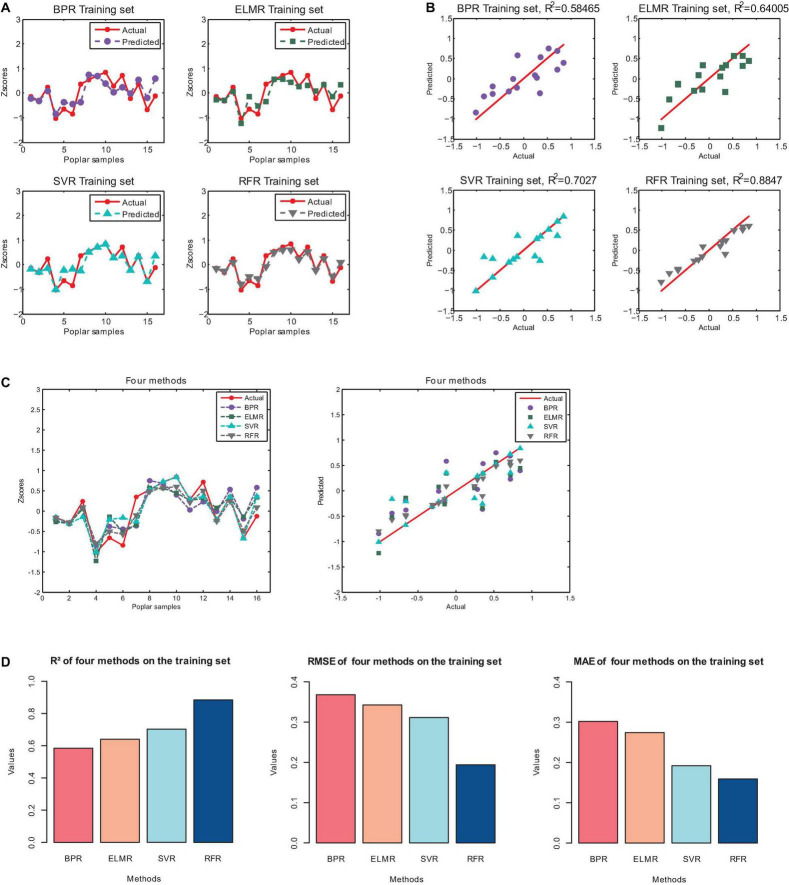
The regression results of the four machine learning methods on the training set. **(A)** Results of Predicted. **(B)** Predicted and actual value. **(C)** Comparison of results of four methods. **(D)** The R^2^, RMSE and MAE of the four machine learning methods on the training set.

Four machine learning regression methods were used to perform regression prediction on the three screened features (Fv, qN_Fo and RH_S/RH_R). The results obtained on the training set, and the corresponding R^2^, RMSE, and MAE are shown in Figure 6 and Table 6.Figure 6D is a histogram of model evaluation indexes (R^2^, RMSE, and MAE) of four machine learning methods on the training set. The colored columns correspond to the four machine learning methods of BPR, ELMR, SVR, and RFR, respectively. From the first subplot of Figure 6D, it can be noticed that on the training set, the highest R^2^ of the four machine learning methods is random forest regression (RFR). Specifically, from Figure 6B and Table 6, we can observe that the coefficient of determination (R^2^) of RFR is 0.8847. Then, the second one is support vector regression (SVR), the R^2^ is 0.7027. In contrast, the performance of BP neural network regression (BPR) and Extreme learning machine regression (ELMR) methods are relatively poor, and their R^2^ are 0.5847 and 0.6401, respectively. In addition, from Figure 6D, we can get similar results from the performance of RMSE and MAE. Similarly, from Table 6, we can find that the RMSE of the RFR method is the smallest with a value of 0.1940, and at the same time, the MAE of RFR is also the smallest, with a value of 0.1591. Therefore, for the four machine learning methods, the RFR method has the best regression effect. Then, the second is the SVR method. Correspondingly, the prediction effects of ELMR and BPR on the training set are relatively trivial.

**TABLE 6:** The R^2^, RMSE and MAE of the training set.

Methods	BPR	ELMR	SVR	RFR
R^2^	0.5847	0.6401	0.7027	0.8847
RMSE	0.3680	0.3426	0.3113	0.1940
MAE	0.3019	0.2741	0.1920	0.1591

#### Test Set

**FIGURE 7:**
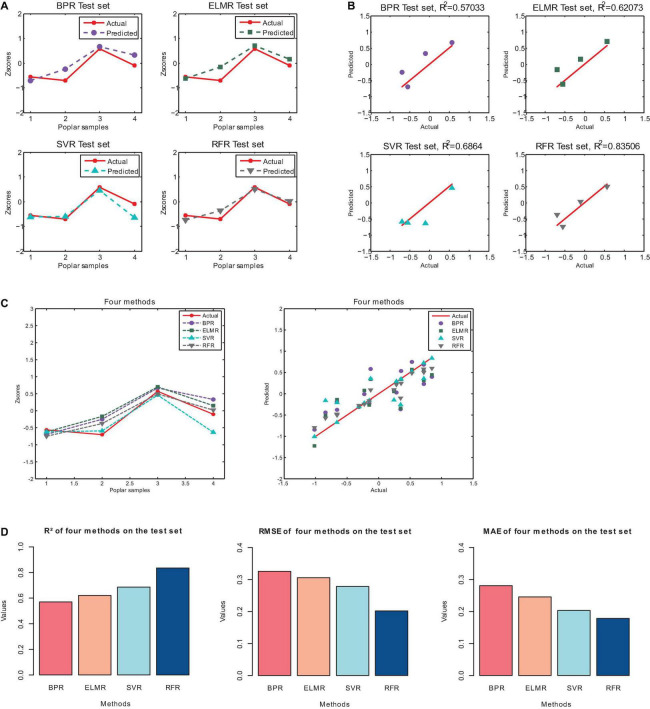
The regression results of the four machine learning methods on the test set. **(A)** Results of Predicted. **(B)** Predicted and actual value. **(C)** Comparison of results of four methods. **(D)** The R^2^, RMSE and MAE of the four machine learning methods on the test set.

Similarly, the results of the four machine learning regression methods on the test set, and the corresponding R^2^, RMSE and MAE are shown in Figure 7 and Table 7.

**TABLE 7:** The R^2^, RMSE and MAE of the test set.

Methods	BPR	ELMR	SVR	RFR
R^2^	0.5703	0.6207	0.6864	0.8351
RMSE	0.3254	0.3057	0.2780	0.2016
MAE	0.2806	0.2456	0.2032	0.1782

Figure 7D is a histogram of model evaluation indexes (R^2^, RMSE, and MAE) of four machine learning methods on the test set. The colored columns correspond to the four machine learning methods of BPR, ELMR, SVR, and RFR, respectively. As shown in Figure 7D, random forest regression (RFR) has the highest R^2^ for the four machine learning methods on the test set. In addition, from Figure 7B and Table 7, we can observe that the R^2^ of RFR is 0.8351. Then, the second one is SVR, the R^2^ is 0.6864. The third and fourth are ELMR and BPR, their performance is relatively poor, and the corresponding R^2^ are 0.6207 and 0.5703, respectively. Besides, from Figure 7D and Table 7, on the test set, the smallest root mean square error (RMSE) is RFR, followed by SVR and other methods. Similar results appear on the mean absolute error (MAE). Consequently, our results show that not all machine learning algorithms can show high accuracy. The best performance on the test set is RFR, followed by SVR. Then, the third and fourth are ELMR and BPR. This result is consistent with the training set.

In summary, according to the results of the training set and the test set, for the flood resistance of poplar, the best prediction effect of the four machine learning methods is random forest regression (RFR), and the second one is support vector regression (SVR). By contrast, the performance effects of BP neural network regression (BPR) and Extreme learning machine regression (ELMR) methods are poor. The prediction accuracy from high to low is RFR > SVR > ELMR > BPR. Hence, when predicting the flood resistance of poplar, random forest regression (RFR) and support vector regression (SVR) can be used first, and RFR can be given more consideration.

## Discussion

**FIGURE A1:**
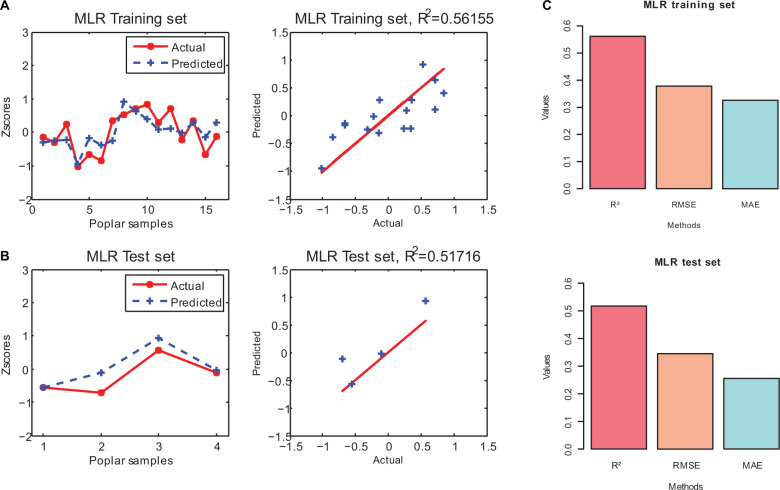
Results of multiple linear regression.

Machine learning is a field of artificial intelligence (AI). Compared with traditional statistical models, machine learning has higher performance, and at the same time, its complexity is relatively lower (Mekanik et al., 2013). In fact, before establishing the regression model of machine learning, we performed multiple linear regression (MLR) on the five variables (Fv, qN_Fo, Fm, H_2_OS/H_2_OR, and RH_S/RH_R) selected by the significance testing and correlation analysis. However, the results show that the coefficients of determination (R^2^) of MLR on the training set and test set are 0.5616 and 0.5172, respectively. The regression results are shown in Appendix Figure A1. Many studies have compared machine learning models with traditional statistical models (Aertsen et al., 2010; Rezaeianzadeh et al., 2014; Idowu et al., 2016; Johnson et al., 2016; Wang and Srinivasan, 2017). In most cases, machine learning models are better than traditional statistical models, such as linear regression. The model and the variables are not linearly related in most situations, and the variables involved are also multivariate. Therefore, more and more fields have begun to use machine learning algorithms. Even so, while machine learning has many advantages, it also has limitations. For example, many machine learning models lack interpretability and are prone to overfitting. For this reason, these problems still need to be considered in practical applications.

The risk of resisting flood disasters can be mainly divided into two aspects. One is to directly predict flood disasters in the risk areas, and take preventive measures before the disaster occurs, such as transferring personnel and valuable finances. Generally speaking, the key variables that need to be considered in flood forecasting include 25 factors such as water level, river flood, soil moisture, and rainfall (Maier et al., 2010). Among these key variables affecting flood forecasting, rainfall and the spatial examination of the hydrologic cycle have the most significant effects (Nourani and Komasi, 2013). Although many studies have predicted the risk of flooded areas (Sampson et al., 2015; Tehrany et al., 2015; Wang et al., 2015; Darabi et al., 2019), this method still cannot essentially eliminate the impact of flood disasters. At the same time, it is relatively difficult to predict flood disasters. Thus, people have to consider another method to resist flood disasters. Another way to resist the impact of flood disasters is mainly through building dams and afforestation. The key to afforestation is to understand the waterlogging resistance mechanism of plants. The research on the waterlogging resistance mechanism of plants mainly focuses on exploring the ways for plants to resist flood stress (Wang et al., 2013, 2021; Najeeb et al., 2015; Duy et al., 2016). These studies have analyzed the waterlogging resistance mechanism from the molecular level of proteins and metal ions. However, there are few studies on waterlogging resistance prediction, and a complete system is still lacking. Xie and Shen (2021) used the SVR method in machine learning to predict the waterlogging resistance of poplar. However, there are still some limitations in their studies, such as chlorophyll fluorescence features that have not been considered. Compared with the previous research, we considered more accurate feature parameters and more kinds of machine learning methods. Additionally, we improved the prediction system of poplar resistance to waterlogging and added two quantitative definitions of waterlogging resistance evaluation indexes, which has made considerable improvements.

This study used machine learning methods to predict the flood resistance of poplar. First, three indicators of flood resistance were defined and evaluated. Then, the data was processed, and feature selection and modeling evaluation were implemented. The whole process is intuitive and specific, which has perfected the research system of waterlogging tolerance prediction to a considerable extent, and at the same time, it has also promoted the research on the mechanism of waterlogging tolerance. This study helps researchers to screen out poplar varieties with strong waterlogging tolerance during the poplar sapling period. It can further cultivate high-quality poplar saplings to achieve the purpose of precise flood resistance. The results of the experiment show that the machine learning algorithm shows high accuracy in predicting the flood resistance of poplar, especially the random forest regression (RFR) and support vector regression (SVR) methods. The final result has certain practical value. In practical applications, these two algorithms can be used first. However, it must be mentioned that although 160 poplar samples were used throughout the experiment, only 20 poplar varieties were actually used for regression analysis. In addition, in the regression analysis, 80 poplar samples from the experimental group were used and averaged according to varieties. Since the waterlogging tolerance of different individuals may be quite different, the final result may deviate from the actual situation. But within the allowable range of error, our research mainly provides a way of predicting waterlogging tolerance and improving the system for predicting waterlogging tolerance. Future research can consider more poplar varieties to improve the universality and stability of the method. In addition, the quantitative relationship of poplar varieties’ impact on flood disasters can be considered. In a word, this research has great theoretical value and practical significance, and the proposed method can meet the actual engineering needs in a considerable range.

## Conclusion

To predict the flood resistance of poplar, the author first analyzed the differences between the three evaluation indexes of flood resistance. Then, the final evaluation index of waterlogging tolerance was determined, and outliers were eliminated. For the selection of feature parameters, the first screening was carried out according to the significance test and correlation analysis, and then the three methods of hierarchical clustering, Lasso, and stepwise regression were adopted to screen the features for the second time. The selected features are interpretable and promote the understanding of poplar’s waterlogging resistance mechanism. Finally, four machine learning methods were used to predict and evaluate the flood resistance of poplar. The results show that the random forest regression and support vector regression methods are more precise. Nevertheless, it must be pointed out that there are only four groups of experiments and controls for each variety. Due to sample differences and randomness, the final result may deviate from the actual situation. Future research can consider more poplar species and sample sizes to improve the versatility and stability of the method.

This research has perfected the prediction system of plant resistance to waterlogging, and has important value for accurate flood resistance and scientific seedling selection. Meanwhile, it has also made a great contribution to a better understanding of the mechanism of waterlogging tolerance. The analysis process of this paper is clear and repeatable. When considering the features related to the flood resistance of poplar, the photosynthesis features, chlorophyll fluorescence features, and environmental features are comprehensively considered. After data processing, feature selection, and other operations, the machine learning models were used to predict the flood resistance of poplar. Finally, the regression results show that the random forest regression (RFR) and support vector regression (SVR) methods have high accuracy. On the test set, the coefficients of determination (R^2^) of the two methods are 0.8351 and 0.6864, respectively, the root mean square errors (RMSE) are 0.2016 and 0.2780, and the mean absolute errors (MAE) are 0.1782 and 0.2031. Based on the above conclusions, our research shows that combining photosynthesis, chlorophyll fluorescence, and environmental variables before flooding experiments, modeling and prediction of machine learning methods against waterlogging can achieve high accuracy, which is suitable for actual engineering problems.

## Data Availability Statement

The raw data supporting the conclusions of this article will be made available by the authors, without undue reservation.

## Author Contributions

XX, XZ, JS, and KD participated in the conception and design of this research and revised the manuscript. XZ and KD carried out experiments and organized the database. XX and JS performed the statistical analysis and proposed the methodology. XX implemented visualization and wrote the manuscript. All authors contributed to the article and approved the submitted version.

## Conflict of Interest

The authors declare that the research was conducted in the absence of any commercial or financial relationships that could be construed as a potential conflict of interest.

## Publisher’s Note

All claims expressed in this article are solely those of the authors and do not necessarily represent those of their affiliated organizations, or those of the publisher, the editors and the reviewers. Any product that may be evaluated in this article, or claim that may be made by its manufacturer, is not guaranteed or endorsed by the publisher.

## Appendix A

**TABLE A1:** The specific meanings and correlation coefficients of 26 features.

Features	Specific meaning	Unit
AHs/Cs	Ball-Berry parameter	Dimensionless
Cond	Conductance to H_2_O	mol H_2_O m^–2^ s^–1^
CndCO_2_	Total conductance to CO_2_	mol CO_2_ m^–2^ s^–1^
CO_2_S	CO_2_ concentration on Sample cell	μmol CO_2_ mol^–1^
CO_2_S/CO_2_R	CO_2_ concentration on Sample cell/CO_2_ concentration on Reference cell	Dimensionless
C_2_sfc	CO_2_ concentration on Leaf Surface	μmol CO_2_ mol^–1^
Ci_Pa	Intercellular CO_2_ partial pressure	Pa
Ci/Ca	Intercellular CO_2_/Ambient CO_2_	Dimensionless
CndTotal	Total conductance to water vapor	mol H2O m^–2^ s^–1^
CTleaf	Computed leaf temperature	°C
Fo	Minimal fluorescence (dark)	bit
Fm	Maximal fluorescence (dark)	bit
Fv	Variable fluorescence	bit
H_2_OS	H_2_O concentration on Sample cell	mmol H_2_O mol^–1^
H_2_OS/H_2_OR	H_2_O concentration on Sample cell/H_2_O concentration on Reference cell	Dimensionless
H_2_O_i_	Intercellular H_2_O concentration	mmol H_2_O mol^–1^
H_2_Odiff	Difference between Intercellular H_2_O and Sample cell H_2_O	mmol H_2_O mol^–1^
Photo	Photosynthetic rate	μmol CO_2_ m^–2^ s^–1^
PARabs	Absorbed Photosynthetically active radiation	μmol m^–2^ s^–1^
PhiCO_2_	Quantum yield corresponding to CO_2_ assimilation rate	Dimensionless
qN_Fo	Non-photochemical quenching (Calculated by Fo)	Dimensionless
RH_S	Relative humidity in the sample cell	%
RH_S/RH_R	Relative humidity on Sample cell/Relative humidity on Reference cell	Dimensionless
SVTleaf	Saturated vapor pressure calculated by leaf temperature	Pa
Trans	Transpiration rate	mol H_2_O m^–2^ s^–1^
VpdL	Vapor pressure deficit based on Leaf temperature	kPa
